# Cell-specific expression of artificial microRNAs targeting essential genes exhibit potent antitumor effect on hepatocellular carcinoma cells

**DOI:** 10.18632/oncotarget.3302

**Published:** 2015-01-21

**Authors:** Chenyu Mao, Hao Liu, Ping Chen, Jingjia Ye, Lisong Teng, Zhenyu Jia, Jiang Cao

**Affiliations:** ^1^ Clinical Research Center, The Second Affiliated Hospital of Zhejiang University School of Medicine, Hangzhou, Zhejiang, P. R. China; ^2^ Institute of Occupational Diseases, Zhejiang Academy of Medical Sciences, Hangzhou, Zhejiang, P. R. China; ^3^ Sir Run Run Shaw Institute of Clinical Medicine, Zhejiang University, Hangzhou, Zhejiang, P. R. China; ^4^ Cancer Center, The First Affiliated Hospital of Zhejiang University School of Medicine, Hangzhou, Zhejiang, P. R. China

**Keywords:** Hepatocelluar Carcinoma, Gene Therapy, Artificial MicroRNA, RNA Interference

## Abstract

To achieve specific and potent antitumor effect of hepatocyte carcinoma cells, replication defective adenoviral vectors, namely rAd/AFP-amiRG, rAd/AFP-amiRE and rAd/AFP-amiRP, were constructed which were armed with artificial microRNAs (amiRs) targeting essential functional genes glyceraldehyde-3-phosphate dehydrogenase, eukaryotic translation initiation factor 4E and DNA polymerase α respectively under the control of a recombinant promoter comprised of human α-fetoprotein enhancer and basal promoter. The AFP enhancer/promoter showed specific high transcription activity in AFP-positive HCC cells Hep3B, HepG2 and SMMC7721, while low in AFP-negative cell Bcap37. All artificial microRNAs exhibited efficient knockdown of target genes. Decreased ATP production and protein synthesis was observed in rAd/AFP-amiRG and rAd/AFP-amiRE treated HCC cells. All three recombinant adenoviruses showed efficient blockage of cell cycle progression and significant suppression of HCC cells in vitro. In nude mice model bearing Hep3B xenograft, administration of rAd/AFP-amiRG showed potent antitumor effect. The strategy of tumor-specific knockdown of genes essential for cell survival and proliferation may suggest a novel promising approach for HCC gene therapy.

## INTRODUCTION

Cancer is one of leading causes of human death worldwide. Liver cancer ranks the second in men and the sixth in women among cancer-caused deaths [1]. Hepatocellular carcinoma (HCC) is the main type of primary liver cancer. Though efforts have been made to improve the outcomes in surgical, chemotherapeutic and radiotherapeutic treatment of HCC, the mortality still remains high. Many novel strategies are being proposed and investigated for their potential in HCC treatment including those through biological means such as gene therapy, which has shown the promising potentiality in cancer therapeutic interventions [2,3].

Multiple genetic and epigenetic alterations are involved in the development and progression of malignancies such as inactivation of tumor suppressors by mutation or down-regulation, activation of oncogenes by amplification, mutation or up-regulation, and other dysregluation of genes responsible for cell growth and proliferation, apoptosis, metastatic potential, and abnormal levels of noncoding regulatory microRNAs. Various approaches of gene therapy for cancer have been extensively investigated for their potential in inhibition/reversal of malignant phenotypes of cancer cells such as uncontrolled proliferation, evasion of apoptosis, invasion and metastasis, angiogenesis, etc. by aiming at individual abnormal gene in tumor cells. However, malignancy is a final consequence of multiple abnormalities at multiple levels and via multiple pathways, with cross-interactions or cross-talks, correction of individual abnormality may not lead to the expected eradication of malignancies. One simple example is that it was reported that efficient inhibition of expression of the anti-apoptotic gene bcl-xl in mesothelioma cells did not lead to expected efficient apoptosis, because the expression of another anti-apoptotic gene bcl2 was unexpectedly up-regulated when bcl-xl expression was inhibited [4]. We also failed to induce efficient apoptosis in some other tumor cells when bcl2 expression was inhibited efficiently (unpublished data).

In seeking for a new approach for more effective HCC gene therapy, we proposed that specifically blocking the fundamental metabolic processes including energy supply, protein synthesis and DNA replication in HCC cells might inhibit the growth and proliferation of cancer cells and therefore achieve potent therapeutic effects. Indispensable genes in these fundamental processes can be good candidate targets.

The house-keeping gene glyceraldehyde-3-phosphate dehydrogenase (GAPDH) is a major enzyme in the energy-generating process glycolysis, which is more important in energy supply of cancer cells than in that of normal cells because cancer cells metabolize glucose mainly through the glycolytic pathway and depend far less on oxidative phosphorylation [5]. Most cancer cells exhibit increased glycolysis and use this metabolic pathway for generation of ATP as a main source of their energy supply and GAPDH may be a potential target for cancer intervention [6]. Rapid proliferation of cancer cells requires large quantities of new protein synthesis, and the eukaryotic translation initiation factor 4E (eIF4E), a key regulator of mRNA export and an important component of the complicate and delicate protein synthesis machinery, is often elevated in many cancers [7]. DNA replication is also needed for the proliferation of cancer cells, and DNA polymerase α, one of three major eukaryotic replicases, is responsible for the initiation of DNA replication at origins and during lagging-strand synthesis of Okazaki fragments [8]. Therefore, GAPDH, eIF4E and DNA polymerase α might be suitable targets for simultaneous inhibition to restrain growth of hepatocellular carcinoma cells by interrupting of energy supply, protein synthesis and DNA replication and ultimately triggering cell death.

RNA interference (RNAi), an important post-transcriptional gene silencing process by targeting specific mRNA transcripts for degradation or translational repression in a wide spectrum of organisms, has been rapidly developed into one potent therapeutic technology for cancer by affording researchers a powerful implement to block overexpressed genes in cancer cells with sequence specificity. Various technologies employing siRNAs, shRNAs and miRNAs have been adopted in a number of cancer gene therapy studies [9,10]. miRNA has been widely used due to its higher post transcriptional processing efficiency and safety over both siRNAs and shRNAs [11,12]. miRNAs are expressed as larger primary transcripts (pri-miRNAs) and excised to generate intermediates, pre-miRNAs, which are subsequently exported to cytoplasm and diced into functional miRNAs. Naturally occurred pri-miRNAs may contain one to six pre-miRNAs in a single transcript. Tandem arrays of repeats of one miRNA or different miRNAs driven by one promoter showed enhanced efficiency of target gene repression or to multigene suppression [13,14].

Most natural miRNA transcriptions are driven by Pol II promoters, therefore artificial miRNA (amiRNA) can be controlled by tissue-specific promoters belonging to Pol II promoters for specific expression in tumor cells. The oncofetal glycoprotein, α-fetoprotein (AFP), is almost undetectable in normal adult liver and other tissue cells, but is expressed abundantly in fetal liver and primary liver cancer cells. AFP is a HCC specific biomarker and AFP promoter has been used in many gene therapy studies targeting HCC [15,16]. As most tissue-specific promoters, AFP promoter has lower transcriptional activity. Previous studies showed the presence of an upstream transcriptional enhancer with strong tissue-specific activity, and transcriptional regulatory element containing this enhancer has been demonstrated to improve AFP promoter activity efficiently when being employed to target to HCC cells, limiting toxicity to surrounding normal cells [17,18].

In this study, we tested the hypothesis that targeted knock down of essential genes for cell survival and proliferation would efficiently kill HCC cells. The strategy of tandem repeats of artificial miRNAs against GAPDH, eIF4E and DNA polymerase α under the transcriptional control of AFP enhancer/promoter were applied in our study.

## RESULTS

### Specific transcriptional activity of AFP enhancer/promoter in HCC cells

**Figure 1:**
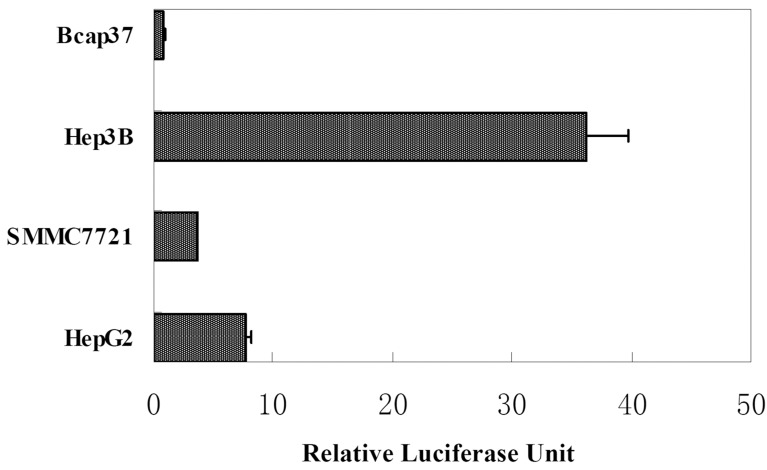
Relative luciferase activity of AFP enhancer/promoter in cell lines Luciferase reporter plasmid pGL4.10/AFP enhancer/promoter was cotransfected with pGL4.74 and luciferase assay was performed 24 hours post transfection. Relative luciferase units (RLUs) represent the Firefly luciferase activity in each sample after normalization by Renilla luciferase activity.

The AFP enhancer and basal promoter cloned by PCR were ligated to form a recombinant AFP transcriptional regulatory element AFP enhancer/promoter. The transcriptional activities of AFP enhancer/promoter in different cell lines were measured by Dual-Luciferase Reporter Assay System. Plasmid pGL4.74, expressing Renilla luciferase driven by non-tissue-specific TK promoter, was used as internal control to normalize transfection efficiency. Promoter-less plasmid pGL4.10 was used as negative control. Firefly luciferase activities in different samples were expressed as relative luciferase units (RLUs) after normalization to internal control. In all AFP-positive HCC cell lines, Hep3B, HepG2 and SMMC7721, AFP enhancer/promoter showed specific transcription activities with luciferase activities 42-, 9- and 5-folds of that in AFP- negative Bcap37 respectively, as shown in Figure 1.

### Construction of AFP promoter-driven adenoviral vectors containing amiRNAs

**Figure 2:**
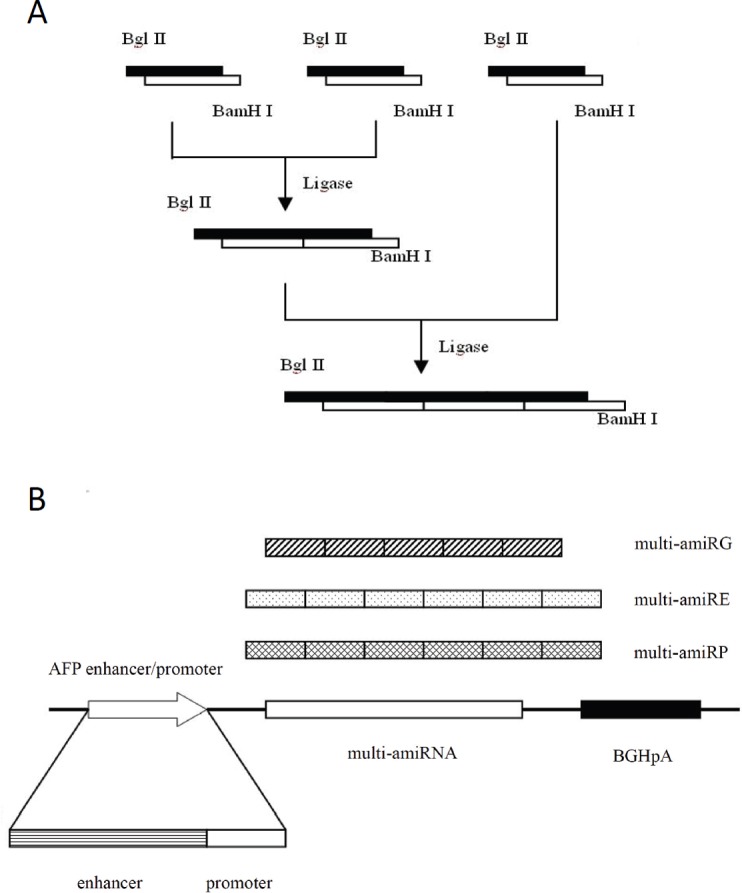
The schematic diagram of amiRNA cloning A: Multi-hairpin amiRNAs were obtained by ligation of individual amiRNAs in head-over-end manner by compatible adhesive ends generated by restriction endonucleases *Bgl* II and *Bam*H I. B: The expression cassette of amiRNAs. Recombinant AFP enhancer/promoter controls the specific transcription initiation of each multi-hairpin amiRNA, followed by bovine growth hormone polyadenylation sequence for termination of transcription.

The expression cassette inserted into the adenoviral vector was depicted in Figure 2. The recombinant AFP transcriptional regulatory element was utilized to control the expression of tandem repeat of amiRNAs targeting GAPDH, eIF4E or DNA polymerase α respectively to restrict the expression of amiRNAs in HCC cells. The sequences of the amiRNAs used in this study were listed in Table 1. High titers of recombinant adenoviruses rAd/AFP-amiRG (with amiRNA targeting GAPDH), rAd/AFP-amiRE (with amiRNA targeting eIF4E) and rAd/AFP-amiRP (with amiRNA targeting DNA polymerase α) were obtained using the AdMax^TM^ System.

**Table 1:** Sequence list of amiRNAs

Name	Sequence
**amiRG**	5′-*AGATCT*GATCCAAGAAGGTATATTGCTGTTGACAGTGAGCGCGCTCATTTCCTGGTATGACAATAGTGAAG CCACAGATGTATTGTCATACCAGGAAATGAGCTTGCCTACTGCCTCGGACTTCAAGGGCTACGAT*GGATCC*-3′
**amiRE**	5′-*AGATCT*GATCCAAGAAGGTATATTGCTGTTGACAGTGAGCGCGGAGCACTAGTTTGATTATTATAGTGAAG CCACAGATGTATAATAATCAAACTAGTGCTCCATGCCTACTGCCTCGGACTTCAAGGGCTACGAT*GGATCC*-3′
**amiRP**	5′*AGATCT*GATCCAAGAAGGTATATTGCTGTTGACAGTGAGCGACCAATTTAG AGTTCATCATTATAGTGAAGC CACAGATGTATAATGATGAACTCTAAATTGGGTGCCTACTGCCTCGGACTTCAAGGGCTACGAT*GGATCC*-3′

### Efficient HCC-specific knockdown of target genes by amiRNAs

**Figure 3:**
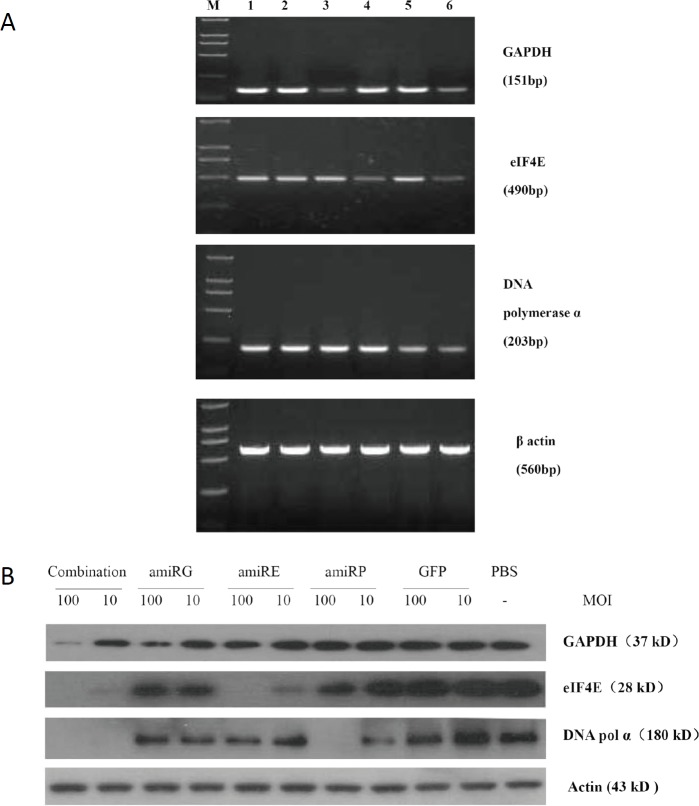
Knock down efficiency of amiRNAs on target genes A: RT-PCR detection of each target gene mRNA level 48 hours after treatment of Hep3B cells with recombinant adenoviruses at MOI 50. M: DL2000 molecular marker, lane 1: PBS control, lane 2: rAd-GFP treatment, lane 3: rAd/AFP-amiRG treatment, lane 4: rAd/AFP-amiRE treatment, lane 5: rAd/AFP-amiRP treatment, lane 6: combination treatment of recombinant adenoviruses with three amiRNAs. B: Western blot detection of protein level of each target gene 48 hours after treatment of Hep3B cells with recombinant adenoviruses at MOI 100 and 10. GAPDH, eIF4E and DNA polymerase α detected by Western blot.

High titer recombinant adenoviruses were prepared to infect cultured cells and RT-PCR and Western blot analyses were performed to evaluate the knockdown efficiency of target genes at mRNA and protein levels respectively. As shown in Figure 3, both mRNA and protein levels of GAPDH, eIF4E and DNA polymerase α in Hep3B cells infected with rAd/AFP-amiRG, rAd5/AFP-amiRE or rAd/AFP-amiRP were significant lower than those in cells infected with rAd/GFP or non-infected cells. Moreover, when Hep3B cells were simultaneously infected with rAd/AFP-amiRG, rAd/AFP-amiRE and rAd/AFP-amiRP, mRNA and protein levels of target genes were also down-regulated significantly. No down-regulation was observed for β-actin, which was used as an internal control. These results indicated that amiRNAs we used in this study could inhibit gene expression at both transcriptional and post-transcriptional levels. AFP enhancer/promoter regulated amiRNA expression could efficiently inhibit the expression of target genes in AFP-positive HCC cells.

### Recombinant adenoviruses blocked the ATP generation in HCC cells *in vitro*

**Figure 4:**
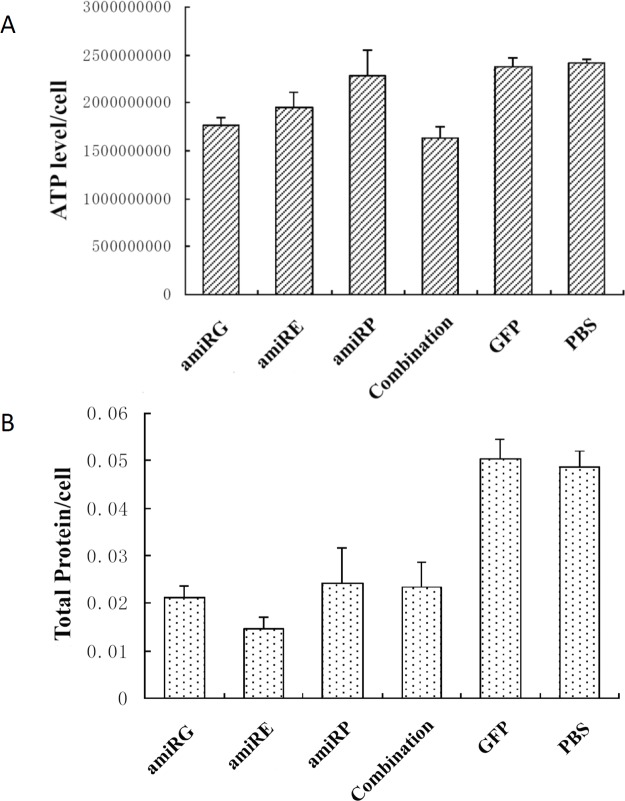
Influence of recombinant adenoviruses on cellular ATP generation and protein synthesis A: ATP levels in Hep3B cells after treatment by recombinant adenoviruses at MOI 50 for 48 hours, adjusted to cell numbers. B: Total protein levels in Hep3B cells after treatment by recombinant adenoviruses at MOI50 for 48 hours, adjusted to cell numbers. Data were presented in mean+SD of triplicates.

GAPDH is a key enzyme in glycolysis which provide the energy for tumor cells under hypoxia condition. Insufficient GAPDH will result in less ATP production. As shown in Figure 4A, the ATP levels in rAd/AFP-amiRG, rAd/AFP-amiRE treated Hep3B cells showed statistical differences when compared to that in rAd/GFP treated or PBS treated control groups, due to the knockdown of GAPDH or GAPDH protein synthesis inhibition caused by efficient eIF4E knockdown. No such phenomenon could be observed in rAd/AFP-amiRP treated Hep3B cells, because the knockdown of DNA polymerase only inhibited the synthesis of DNA and had no influence on GAPDH expression or ATP generation.

### Recombinant adenoviruses influenced the protein synthesis of HCC cells *in vitro*

eIF4E plays a crucial role in cellular protein synthesis by controlling translation initiation. Figure 4B showed that rAd/AFP-amiRE treatment had a remarkable influence on the total protein level of Hep3B cells when compared to rAd/GFP or PBS treatment. The decrease of total protein level in rAd/AFP-amiRG treated Hep3B cells could be caused by insufficient ATP supply in energy-consuming protein synthesis process. The reason for low level total protein exhibited in rAd/AFP-amiRP treated Hep3B cells needs further investigation.

### Recombinant adenoviruses blocked the cell cycle progression of HCC cells

**Figure 5:**
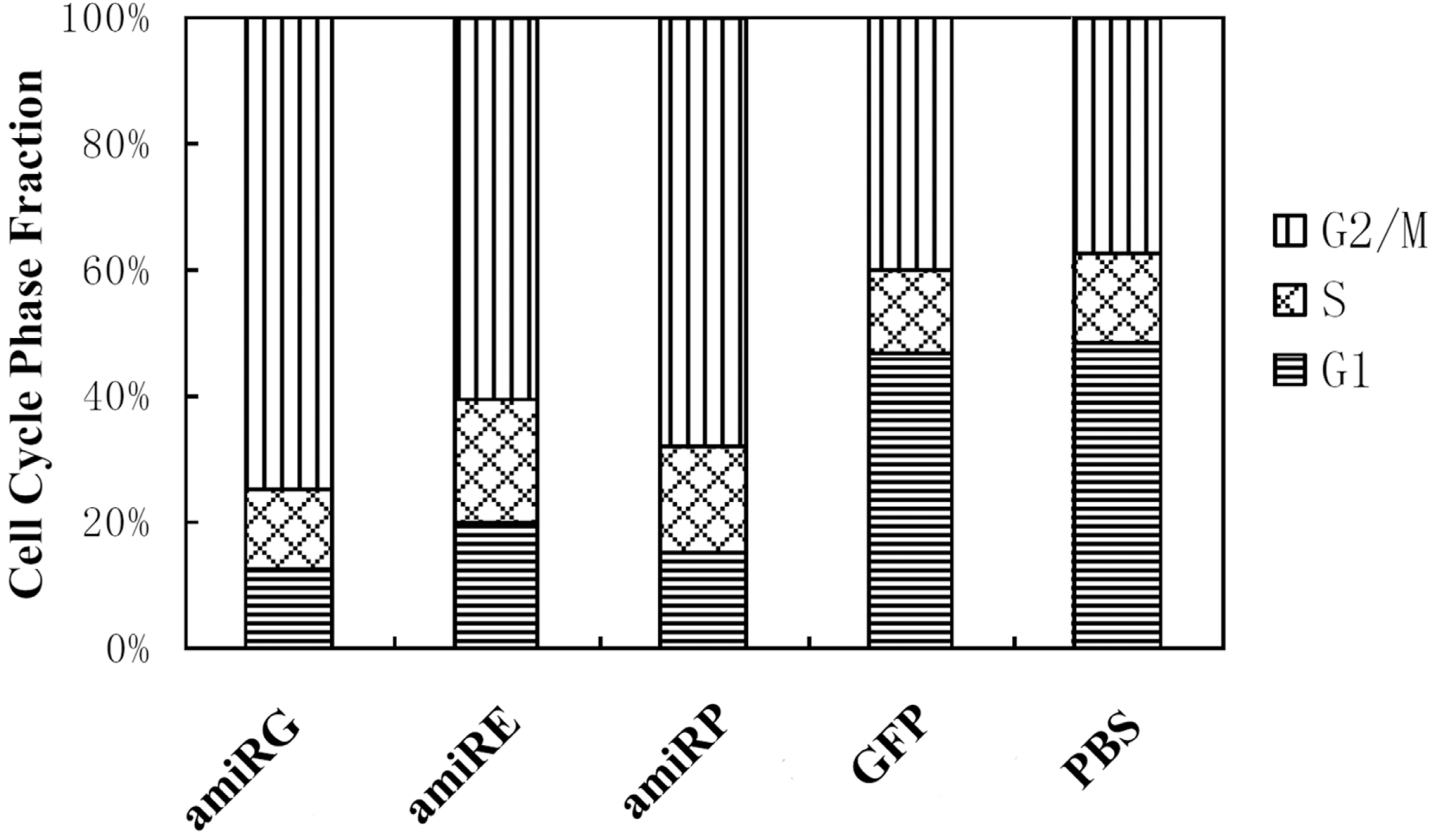
Cell cycle phase fractions of Hep3B cells Hep3B cells were infected by recombinant adenoviruses at MOI 200 for 48 hours and subject to flow cytometer analysis.

As shown in Figure 5, the cell cycle progression of Hep3B cells was blocked at G_2_/M phase by each of the recombinant adenoviruses with amiRNAs, while no such blockage could be observed on cells infected with control virus rAd/GFP or non-infected cells, which suggested that it was the efficient knockdown of GAPDH, eIF4E or DNA polymerase α in Hep3B cells that interfered the progression of cell cycle.

### Recombinant adenoviruses specifically inhibited HCC cells *in vitro*

**Figure 6:**
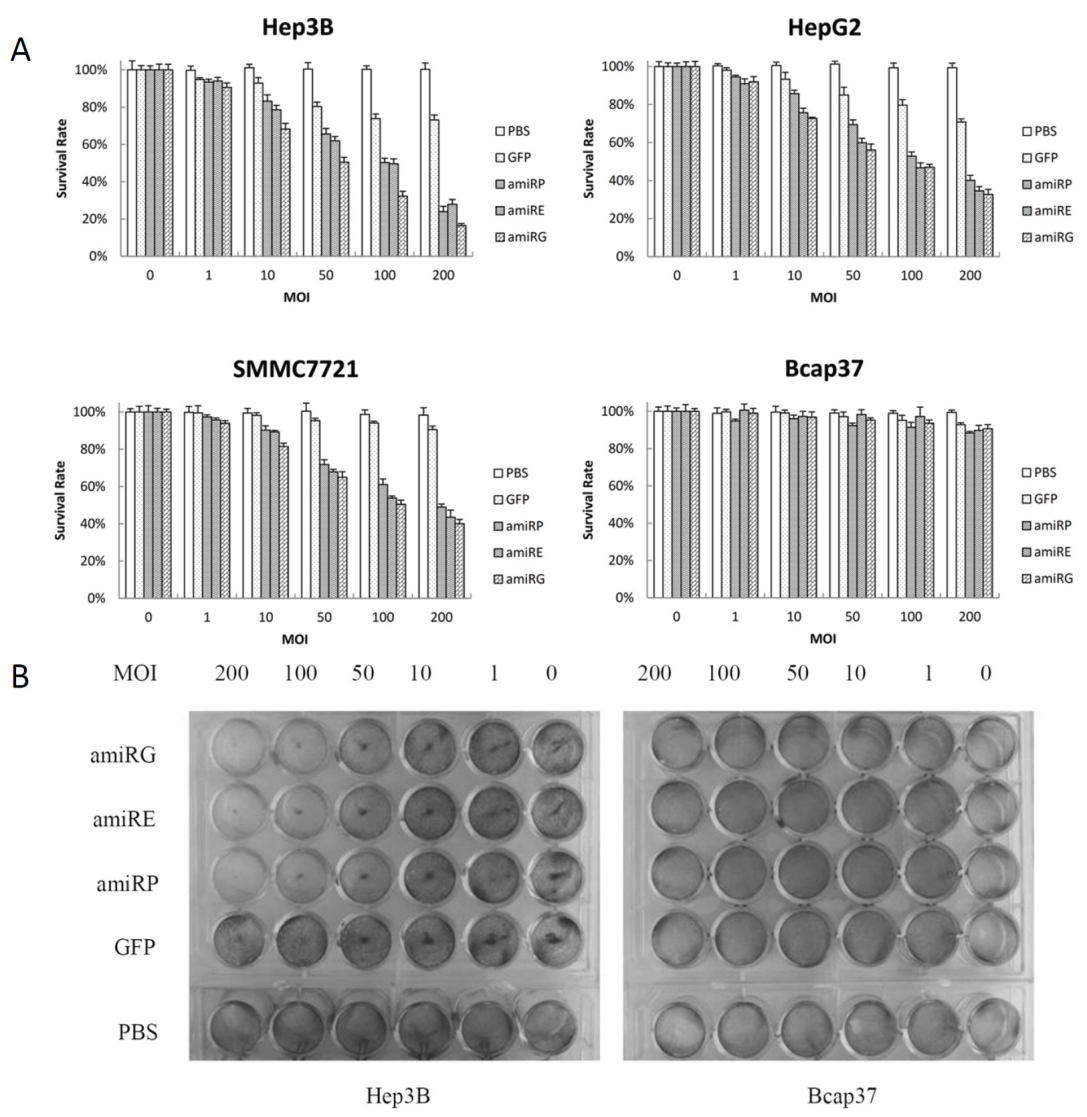
Growth inhibition of different cells infected with recombinant adenoviruses A: MTT assay was performed to exam the viability of cells infected at different MOI (pfu/cell) by recombinant adenoviruses for 4 days. Data were presented as mean±SD of triplicates. B: Crystal violet staining of cells infected by recombinant adenoviruses at different MOIs for 4 days.

Both AFP-positive and -negative cell lines were used to evaluate the selective inhibition of these recombinant adenoviral vectors with AFP modulated amiRNAs. As shown in Figure 6A, after infecting cells with adenovirus for 4 days, the survival rates of AFP-positive cells Hep3B, HepG2 and SMMC7721 were significantly lower than the AFP-negative cell Bcap37 in a MOI-dependent manner, while no inhibition could be observed with rAd/GFP control virus. The crystal violet staining also showed the specific inhibition of Hep3B cells by recombinant adenoviruses as compared to that of Bcap37 cells (Figure 6B).

### *In vivo* antitumor efficacy of rAd/AFP-amiRG

**Figure 7:**
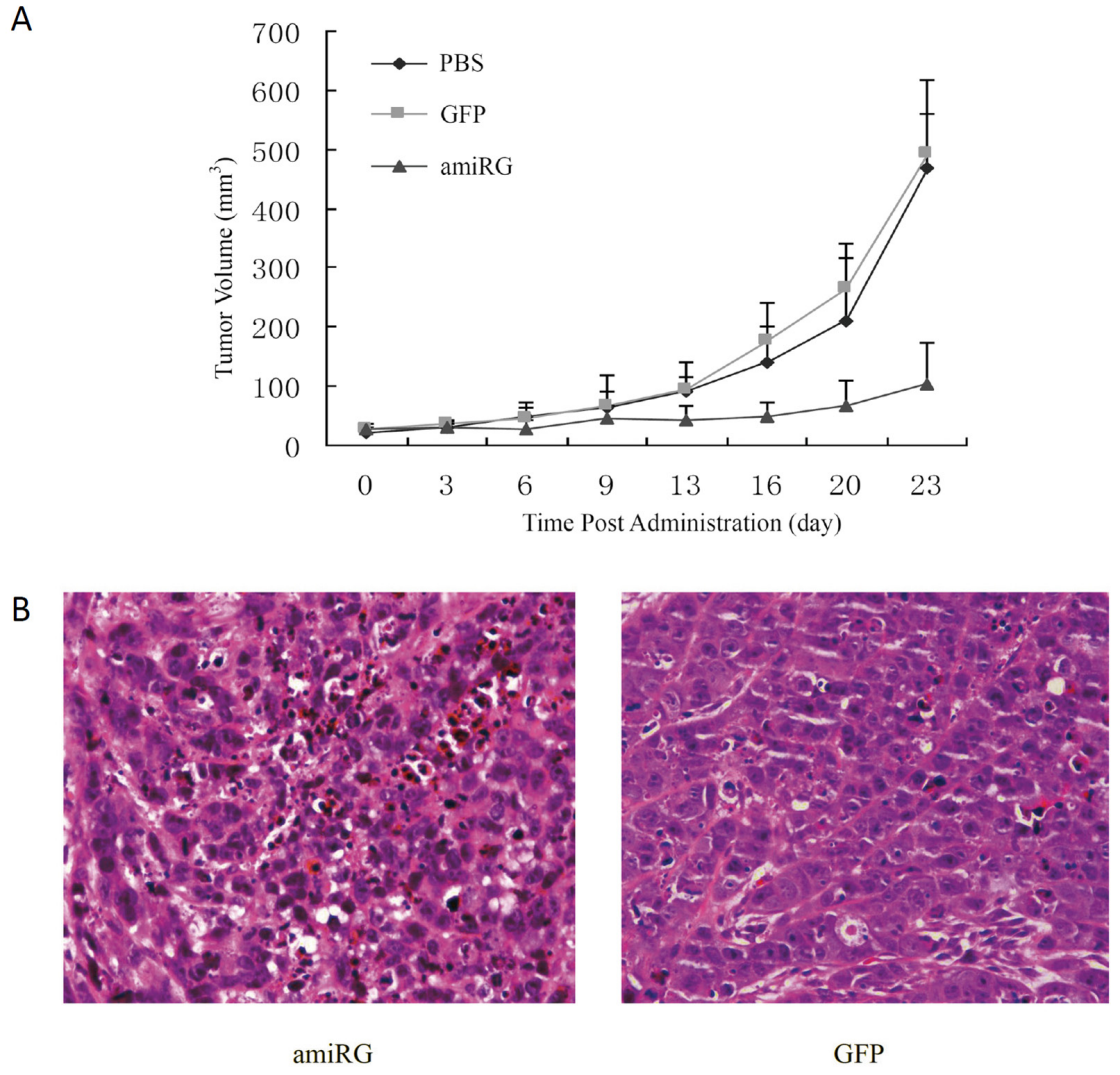
*In vivo* anti-tumor effect of rAd/AFP-amiRG A: Hep3B tumor xenografts in BALB/c nude mice were measured every three days and tumor volume was calculated using the formula V=(lengthxwidth^2^)/2 after intratumoral injection of rAd/AFP-miRG, rAd-GFP or PBS. B: HE staining of tumor xenografts (400X).

*In vivo* antitumor efficacy of rAd/AFP-amiRG was assessed in athymic BALB/C (nu/nu) mice with Hep3B xenograft model. Administration of rAd/AFP-amiRG by intratumoral injection demonstrated potent antitumor efficacy, as shown in Figure 7A. The rAd/GFP control virus showed no significant difference with comparison to PBS administration group. Pathologic examination showed that rAd/AFP-amiRG treatment caused significant destruction of the xenografted tumor (Figure 7B).

## DISCUSSION

Gene therapy, originated in 1960 and conceptualized in 1972, is usually an approach to introduce exogenous DNAs encode functional proteins or therapeutic protein drugs (rather than natural human genes) into individual's cells to correct genetic deficiency caused by defective genes [19]. However, similar to antisense therapy which inhibits endogenous gene expression, broadly RNAi-based therapy is not strictly a form of functional gene therapy but a genetically-mediated therapy. About 2/3 of all gene therapy clinical trials are for the treatment of cancer in the past decades [20]. By now, many cancer gene therapy studies aim at various targets to correct their aberrant expression in tumors, such as inhibition of over-expressed oncogenes, multidrug resistant genes, anti-apoptotic genes, or re-expression of extrinsic normal tumor suppressor genes, introducing tumor suicide genes, anti-angiogenesis genes, apoptosis-inducing genes, immunostimulating genes or in combinations to suppress tumor growth and metastasis, induce apoptosis or mitigating drug resistance, etc. Recently reported works include blocking of a serine/threonine protein kinase Akt (also known as Protein Kinase B, PKB) which plays a key role in multiple cellular processes including glucose metabolism, cell proliferation and cell migration [21]; knock-down of a cell cycle inhibitor BMI-1 (B lymphoma Mo-MLV insertion region 1 homolog) which could enhance drug resistance in B-cell lymphoma cells through the regulation of survivin, a gene often overexpressed in various human cancers to function as an oncogene [22]; silencing Cathepsin B and uPAR (Urokinase type plasminogen activator receptor) overexpressed and involved in tumor angiogenesis, which is vital for tumor progression, migration [23]; suppressing a novel prenylated tyrosine phosphatase PRL-3 which is important in cancer metastasis [24]; inhibiting livin, a new inhibitor of apoptosis protein family [25]; or enhancing the expression of gap junction alpha-1 protein Cx43, an inhibitor of malignant phenotype [26]; increasing the level of DLC1, a tumor growth suppressor gene, frequently silenced in multiple common tumors [27]; strengthening Kringle 5 of human plasminogen, a potent angiogenesis inhibitor [28]; boosting maspin (mammary serine protease inhibitor) functioning to suppress angiogenesis, invasion and metastasis of cancer cells, which could reverse resistance to chemotherapeutic drugs [29]. However, one important issue is that targeting one gene in different tumor cells may result in different antitumor efficacies, or an effective antitumor effect was only limited to some types of tumor, not applicable for other types of tumors, because the alterations of the genes responsible in different tumors are variable.

But all tumor cells are similar in aspects such as energy metabolism, protein synthesis and DNA replication which are indispensable processes for survival and rapid proliferation. Any of these biochemical processes is vital to cell survival and growth. Tumor cells show an increased rate of glucose uptake and utilization [5,30], an accelerated cell division based on more protein synthesis and DNA replication. Therefore, GAPDH, eIF4E and DNA polymerase α are good common targets to be aimed at in all tumors, but which have not been tried till now.

With the progress and widespread application of RNAi technology, artificial miRNAs has showed higher gene silencing efficiency and safety [11,12]. Previous studies have demonstrated that polycistronic transcripts could enhance the efficiency of target gene repression or to achieve linked multi-gene repression [13,14]. Multi-hairpin amiRNAs have demonstrated more powerful on target genes than single-hairpin amiRNAs. In order to get valid transcription and processing in cells, natural miRNA structures are utilized in artificial miRNAs design. amiRNAs based on modified human microRNA 30 (miR-30) could achieve more effective gene silencing than previous short-hairpin RNA (shRNA) [11,13,31]. In this study, we constructed multi-hairpin amiRNAs based on miR-30 to target endogenous genes of GAPDH, eIF4E and DNA pol α to knockdown their expression more effectively.

Specificity is a crucial issue in cancer gene therapy, which is to restrict expression genes of interest only in malignant tissue or cells and prevent expression in surrounding normal tissue or cells to ensure safety. The strategy of utilizing tumor-specific promoter has been widely employed because of its distinctiveness in cancer tissues or cells. In our study, promoter of the HCC tumor marker AFP was used to ensure the HCC specificity. Previous studies showed the presence of strong tissue-specific enhancer activity that is located upstream to the human AFP gene. The AFP enhancer is composed of two components, domain A (−4995 to −3744bp) and domain B (−3744 to −3330bp), both play important roles in up-regulating AFP promoter transcriptional activity in a tissue-specific manner [17,18]. The recombinant AFP transcription element containing AFP enhancer (−4995 to −3330bp) and basal promoter was constructed which showed transcription activity specific in HCC cells in our study.

Under the control of the HCC-specific recombinant AFP promoter, amiRNAs targeting GAPDH, eIF4E and DNA pol α could block major cellular biochemical processes of energy producing glycolysis, protein synthesis and DNA replication only in AFP positive HCC cells, and consequently block cell cycle and ultimately induce apoptosis. Our data showed that all amiRNA constructs used in this study achieved efficient knockdown of each target gene respectively, and achieved potent antitumor effect as well. RAd/AFP-amiRG could effectively inhibit the production of ATP, and also inhibit the protein synthesis. RAd/AFP-amiRE could effectively inhibit the protein synthesis, and also inhibit the ATP production. RAd/AFP-amiRP arrested the cell cycle progression at G_2_/M phase effectively due to blocking the DNA replication which is the genetic basis of cell division, without interfering the energy supply process. In summary, the current work systematically analyzed the tissue specificity, efficiency of gene silencing and antitumor efficacy of the recombinant adenoviral vectors constructed in the study, and provided the basis for exploring a novel effective strategy for HCC gene therapy. This strategy may be extended for other cancer gene therapy by other cancer-specific expression regulatory systems, or for development of other novel therapeutic targets that are essential for the survival, growth and proliferation of cancer cells.

One limitation of the current strategy is that it may be effective only for AFP-positive HCC treatment, and it is not clear if the cancer cells will develop resistance to the treatment by unknown mechanisms. To obtain more potent antitumor efficiency, it will be helpful to combine several strategies in current cancer gene therapy, such as including cytotoxic genes such as recombinant active caspase-3 [32], and adopting oncolytic viral vector.

## MATERIALS AND METHODS

### Materials

Chemicals applied in this study were purchased from Sigma-Aldrich (St Louis, MO, USA) or otherwise specifically stated. PCR primers were synthesised by Invitrogen (Shanghai, P. R. China), restriction enzymes were from New England BioLabs (Ipswich, MA, USA), Taq polymerase, T4 ligase were from Promega Corporation (Madison, WI, USA).

### Cell lines and culture

Human hepatocellular carcinoma cell lines Hep3B and HepG2, human embryo kidney cell line HEK293 were purchased from American Type Culture collection (ATCC, Manassas, VA, USA); human hepatocellular carcinoma cell line SMMC7721, breast carcinoma cell line Bcap37 were obtained from Cell Bank, Shanghai Institutes for Biological Sciences, Chinese Academy of Science (Shanghai, P. R. China). Cells were maintained in Dulbecco's modified Eagle medium (DMEM) or RPMI1640, supplemented with 10% fetal bovine serum. Cells were cultured in 100% humidity, 5% CO_2_ at 37°C. All cell culture products were purchased from GIBCO (Gibco BRL/Life Technologies, Carlsbad, CA, USA).

### Cloning of AFP enhancer/promoter

The 820bp AFP enhancer and 180bp basal promoter were cloned by PCR amplification from human genomic DNA of a healthy donor. Primers for AFP enhancer: upstream: 5′-*AGA TCT C*AG ATT GAA TTA TTT GCC TGT CA-3′, downstream: 5′-*GGA TCC* TAG GAA GTT TTC GCA ATA ATA C-3′; primers of AFP basal promoter: upstream: 5′-*AGA TCT* GCC CCA AAG AGC TCT GTG T-3′, downstream: 5′-*GGA TCC* AAA TCA TGC TGA AAT TCT TTT ATA CTC-3′. The sequences of the cloned AFP enhancer and promoter fragments were verified by DNA sequencing and blast against sequence data in GenBank (accession number NT_006216).

### Generation of AFP enhancer/promoter controlled amiRNA expression recombinant adenoviruses

Sequences of amiRNAs targeting human GAPDH, eIF4E and DNA polymerase α were obtained from RNAi Codex (http://cancan.cshl.edu/cgi-bin/Codex/Codex.cgi) and listed in Table 1. The amiRNAs were cloned by PCR amplifications. Tandem array of amiRNAs (amiRG for amiRNA targeting GAPDH, amiRE for amiRNA targeting eIF4E and amiRP for amiRNA targeting DNA polymerase α) were generated by head-to-end ligation by adhensive ends produced by *Bgl* II and *BamH* I as depicted in Figure 2, and ligated to the 3′-end of AFP enhancer/promoter. The bovine growth hormone polyadenylation sequence from pcDNA3.1(+) plasmid (Life Technologies, Grand Island, NY, USA) was cloned to the 3′-end of amiRNA repeats to form a complete AFP promoter/enhancer controlled amiRNA expression cassette. The amiRNA expression cassettes were inserted into promoter-less plasmid pDC312 (Microbix Biosystems Inc., Mississauga, Ontario, Canada). Recombinant adenoviruses were generated by AdMax^TM^ System (Microbix Biosystems) according to the manufacturer's instructions. In brief, pDC312 plasmids with expression cassettes were co-transfected with packaging plasmid pBHGlox(delta)E1,3Cre into HEK293 cells together by Lipofectamine 2000 (Life Technologies). Primary adenovirus was rescued after homologous recombination in 293 cells and confirmed by PCR. Large scale preparations of the confirmed viruses were purified by CsCl density-gradient centrifugation and titrated for plaque forming unit (pfu).

### Transcriptional activity of AFP enhancer/promoter in various cell lines

The recombinant AFP enhancer/promoter was inserted into the *Bgl* II site of pGL4.10 (Promega Corporation) to yield pGL4.10/AFP enhancer/promoter. The pGL4.74 expressing Renilla luciferase regulated by herpes simplex virus thymidine kinase (TK) promoter was co-transfected with pGL4.10/AFP enhancer/promoter by Lipofectamine 2000 into human HCC cells Hep3B, HepG2, SMMC7721, and breast carcinoma cell Bcap37 at the ratio of 1:100, and served as transfection normalization control. The plasmid pGL4.10 was cotransfected with pGL4.74 as blank control. Luciferase assay was performed 24 hours post transfection using a Dual-Luciferase Reporter Assay Kit (Promega Corporation) according to the manufacturer's instructions. Relative luciferase units (RLUs) of Firefly luciferase activity in each sample were adjusted by Renilla luciferase activity which was used to normalize the transfection efficiency. The transcriptional activities of recombinant AFP enhancer/promoter in different cell lines were compared by RLUs.

### Reverse transcription PCR (RT-PCR)

Hep3B cells were plated in six-well plates at a density of 3×10^5^/well for 24 hours, followed by infection with recombinant adenoviruses at MOI 50. Forty-eight hours after infection, cells were harvested and washed twice with pre-cold PBS. Total RNA was extracted using TRIzol reagent (Life technologies) following the manufacturer's instructions. Two micrograms total RNA was reverse transcribed into cDNA with oligo(dT)_18_ and Superscript First-strand Synthesis Kit (Life technologies), and used as template to amplify GAPDH, eIF4E, DNA polymerase α, and housing keeping gene β-actin. Primer sequences and cycling conditions were listed in Table 2, and PCR products were analyzed on 1.5% agarose gel.

**Table 2:** Primer sequence list for RT-PCR

Name	Primer Sequence	Cycling Protocol	Product Size
GAPDH			
Forward	5′-CTTAGCACCCCTGGCCAAG-3′	95°C 5min;	151bp
Reverse	5′-CGGGGCTCTCCAGAACATC-3′	95°C 30sec, 58°C 45sec, 72°C 45sec (23 cycles); 72°C 10min	
eIF4E			
Forward	5′-ATGGCGACTGTCGAACCGG-3′	95°C 5min;	490bp
Reverse	5′-GCTATCTTATCACCTTTAGC-3′	95°C 40sec, 57°C 40sec, 72°C 70sec (25 cycles); 72°C 10min	
DNA polymerase α			
Forward	5′-TCGCAGTGACAAAACCGAAC-3′	95°C 5min;	203bp
Reverse	5′-TTGGAGCTTCACCGAATCCT-3′	95°C 40sec, 57°C40sec, 72°C 70sec (30 cycles); 72°C 10min	
β-actin			
Forward	5′-AGCAACCGGGAGCTGGTGG-3′	95°C 5min;	560bp
Reverse	5′-CATTTCCGACTGAAGAGTG-3′	95°C 30sec, 58°C 45sec, 72°C 45sec (19 cycles) 72°C 10min	

### Western blot

Hep3B cells were plated in six-well plates at a density of 3×10^5^/well for 24 hours, followed by infection with recombinant adenoviruses at MOI 10 and 100 respectively. Forty-eight hours after infection, cells were harvested and lysed in lysis buffer [1%(v/v) Nonidet P-40, 0.1% SDS, 1 μg/ml Aprotimin, 0.5% sodium desoxycholate, 150 mM NaC1 and 50 mM Tris-HC1, pH 8.0] on ice. Lysates containing 20 μg proteins were separated by 6% (for DNA polymerase α) or 10% (for GAPDH, eIF4E and Actin) sodium dodecyl sulfate polyacrylamide gel electrophoresis (SDS-PAGE). Separated proteins were transferred electrophoretically to PDVF membrane (Millipore, Bedford, MA, USA) in buffer containing 25 mM Tris-HCl (pH 8.3), 192 mM glycine and 20% (v/v) methanol. PDVF membrane was first blocked with 5% (w/v) non-fat milk in TBST containing 20 mM Tris-HCl (pH 7.6), 0.1 M NaCl and 0.1% (v/v) Tween 20 for 1 hour at room temperature. After three washes with TBST for 5 min each time, the membrane was probed with antibodies against GAPDH, eIF4E, DNA polymerase α and Actin (Santa Cruz Biotechnology, Santa Cruz, CA, USA) respectively diluted in 5% bovine serum albumin in TBST for 1 hour at room temperature. The membrane was then washed three times in TBST, and incubated with horseradish peroxidase-conjugated secondary antibodies (Beijing Zhongshan Golden Bridge Biotechnology Co. Ltd., Beijing, P. R. China) diluted by 1:5000 in TBST for 1 hour at room temperature. After three washes with TBST, Immobilon Western Chemiluminescent HRP Substrate (Millipore) was used and bands were visualized by LAS-4000 imaging system.

### MTT assay

Cells were seeded in 96-well plates at a density of 2×10^3^/well in 100μl medium for 24 hours. Recombinant adenoviruses were added at MOIs from 1 to 200 in 100μl and cultured for 4 days. Twenty microliters of MTT (5mg/ml in PBS) was added to medium in each well and incubated for 4 hours. Supernatant was aspirated and 200 μl DMSO was added to each well to dissolve the purple crystals. The optical density (OD) values were read with Model 680 Microplate Reader (Bio-Rad Laboratories, Hercules, CA, USA) at 570 nm. Cell survival rate was calculated using the OD_570nm_ value.

### Crystal violet staining

Hep3B and Bcap37 cells were plated in 24-well plates and infected with recombinant adenoviruses at MOI of 1, 10, 50, 100 and 200 respectively and cultured for 4 days. Cells were washed twice with pre-cold PBS and fixed for 10 minutes with 10% formalin at room temperature, then stained with 0.5% crystal violet solution for 5 minutes. After 3 washes with PBS and dried, the stained plates were recorded by photography.

### Cell cycle analysis

Hep3B cells were infected by recombinant adenoviruses at MOI 200 for 48 hours, and washed twice with ice-cold PBS, fixed with cold 75% ethanol, resuspended in PBS containing 50μg/ml propidium iodide (PI) and 20μg/ml RNase A for 30 minutes at room temperature, and subject to flow cytometer (Beckman Coulter Inc., Brea, CA, USA) for cell cycle analysis.

### ATP assay

Hep3B cells infected with recombinant adenoviruses at MOI 50 for 48 hours were harvested and counted by hemacytometer. Cellular ATP was extracted by 1% trichloroacetic acid, and ATP assay was performed using ENLITEN®ATP Assay System Bioluminescence Detection Kit (Promega) according to manufacturer's instruction with a GloMax™ 20/20 Luminometer (Promega). ATP level was normalized by cell number.

### Protein quantification

Forty-eight hours after infection with recombinant adenoviruses at MOI 50, Hep3B cells were harvested and counted by hemacytometer, and lyzed in lysis buffer (see above in Western blot). Total protein concentration was determined by DC Protein Assay (Bio-Rad Laboratories) according to manufacturer's instruction. To evaluate the inhibiting effect of recombinant adenoviruses on protein synthesis, protein concentration was normalized by cell number.

### Antitumor experiment in nude mice

Experiment was conducted in Experimental Animal Center of Zhejiang Province, P. R. China. Athymic BALB/C (nu/nu) mice were obtained from Shanghai Experimental Animal Center, Chinese Academy of Sciences (Shanghai, P. R. China). Six-week old nude BALB/C mice were inoculated subcutaneously with 5×10^6^ Hep3B cells suspended in 200μl PBS. When tumors were approximately 4-5 mm in diameter measured by calipers, the mice were randomly divided into to three groups with 8 mice for each group: group 1, rAd/AFP-miRG treatment, group 2, rAd-GFP treatment; group 3, PBS control. Recombinant adenoviruses at 2×10^8^pfu/100μl were intratumorally administered three times in 3-day intervals. Tumor sizes were monitored every three days and tumor volume was calculated using the formula V=(length × width^2^)/2. Two days after the last administration, one mouse in each group was randomly selected, sacrificed and tumor tissue was fixed in formalin and embedded in paraffin and used for hematoxylin-eosin staining.

### Statistical analysis

All experiments *in vitro* were repeated three times and results were presented as mean±standard deviation (SD). A P value less than 0.05 was denoted to be statistically significant by Student's t-test.
